# When trust, confidence, and faith collide: refining a realist theory of how and why inter-organisational collaborations in healthcare work

**DOI:** 10.1186/s12913-021-06630-x

**Published:** 2021-06-26

**Authors:** Justin Avery Aunger, Ross Millar, Joanne Greenhalgh

**Affiliations:** 1 Health Services Management Centre, Park House, University of Birmingham, Birmingham, B15 2RT UK; 2 Sociology and Social Policy Department, University of Leeds, Leeds, LS2 9JT UK

**Keywords:** Collaboration, Partnership working, Integration, Healthcare, Improvement, Realist review, Realist synthesis, Context, Programme theory, Implementation

## Abstract

**Background:**

Health systems are facing unprecedented socioeconomic pressures as well as the need to cope with the ongoing strain brought about by the COVID-19 pandemic. In response, the reconfiguration of health systems to encourage greater collaboration and integration has been promoted with a variety of collaborative shapes and forms being encouraged and developed. Despite this continued interest, evidence for success of these various arrangements is lacking, with the links between collaboration and improved performance often remaining uncertain. To date, many examinations of collaborations have been undertaken, but use of realist methodology may shed additional light on *how* and *why* collaboration works, and *whom* it benefits.

**Methods:**

This paper seeks to test initial context-mechanism-outcome configurations (CMOCs) of interorganisational collaboration with the view to producing a refined realist theory. This phase of the realist synthesis used case study and evaluation literature; combined with supplementary systematic searches. These searches were screened for rigour and relevance, after which CMOCs were extracted from included literature and compared against existing ones for refinement, refutation, or affirmation. We also identified demi-regularities to better explain how these CMOCs were interlinked.

**Results:**

Fifty-one papers were included, from which 338 CMOCs were identified, where many were analogous. This resulted in new mechanisms such as ‘risk threshold’ and refinement of many others, including trust, confidence, and faith, into more well-defined constructs. Refinement and addition of CMOCs enabled the creation of a ‘web of causality’ depicting how contextual factors form CMOC chains which generate outputs of collaborative behaviour. Core characteristics of collaborations, such as whether they were mandated or cross-sector, were explored for their proposed impact according to the theory.

**Conclusion:**

The formulation of this refined realist theory allows for greater understanding of *how* and *why* collaborations work and can serve to inform both future work in this area and the implementation of these arrangements. Future work should delve deeper into collaborative subtypes and the underlying drivers of collaborative performance.

**Review registration:**

This review is part of a larger realist synthesis, registered at PROSPERO with ID CRD42019149009.

**Supplementary Information:**

The online version contains supplementary material available at 10.1186/s12913-021-06630-x.

## Introduction

### Background

Global health systems are currently facing unprecedented pressures from a multitude of concurrent events, including limited financial resource, an ageing population, unwarranted variation in healthcare quality, and COVID-19 [1–3]. Of course, there is no ‘magic bullet’ with which to tackle these ongoing issues, however, one solution has become a focus of current planning in the United Kingdom (UK) and elsewhere – that of ‘partnering’, or collaborating between organisations [1, 4]. Such collaborations are purported to foster improved productivity, reduce variation in care quality, and improve financial and clinical sustainability [4]. These arrangements can be both intra- and inter-sectoral, and can take the form of strategic alliances, joint ventures, clinical networks, Vanguard initiatives of various forms, buddying arrangements, mergers, and Integrated Care Systems (ICS’), among others [5]. While defined differently by many authors, common to the definitions of partnering and collaborating is the notion of working together to achieve benefits that would otherwise not be attainable alone [6, 7].

Inter-organisational collaboration is often touted by policymakers as one of the means for solving the ‘wicked issues’ that contemporary health systems are facing. However, it is not always clear how collaborating will solve underlying issues such as financial pressures [1, 7]. Likewise, the process of collaborating itself has its own set of complexities and challenges which require significant time and resource to overcome. These include the impact of historical relationships between partnering organisations, the difficulty of rising above conflicts, building trust, and navigating complex and often contradictory regulatory environments [6, 8, 9]. Inter-organisational collaborations require significant time to establish and work to maintain, and as such it is not surprising that setting up collaborations often results in temporary drops to organisational performance and a lack of attainment of the benefits sought [10–12].

### Rationale for study

A variety of theoretical contributions have been made towards improving understanding of inter-organisational collaborations in healthcare, however, until now, few have attempted to turn a realist lens on the phenomenon [13–18]. For example, Hudson and Hardy (2002) tackled *what* determines a successful partnership, such as a local history of partnership working, effective monitoring and reviewing of organisational learning, having a shared vision, and development and maintenance of trust through behaviours and attitudes such as ‘fairness’, openness and honesty, sacrifice, and accountability. While useful to know ‘what’ leads to successful partnering, it is also important to explore *how* and *why* these features enable collaborations to be successful. *Why* does shared vision aid in accomplishing the aims of a collaboration? *How* does collaboration lead to improved performance?

Realist approaches are valuable to answer these questions and can help to target and adapt partnering approaches to local circumstances. Those who have turned a realist lens to inter-organisational collaborations include Jagosh et al. (2012) with their investigation of participatory research initiatives, and Rycroft-Malone, Burton, Wilkinson, et al. (2016) with their realist review of a particular subtype of healthcare collaboration in the UK. Likewise, Zamboni et al. (2020) have conducted a review drawing on a realist understanding of context, which sought to look at ‘quality improvement collaboratives’ in healthcare [19]. However, other than our initial realist review [18], none have yet attempted to tackle the wider topic of inter-organisational collaborations in healthcare. Here, we have employed a realist review methodology to synthesise a range of studies and develop, test and refine a robust theory of how inter-organisational collaborations in healthcare work, to what extent, why, and in what circumstances. A starting point of realist review is identifying the ideas and assumptions underlying how programmes or interventions work, known as programme theories. Realists also work with the premise that programmes are never universally successful; rather, how they work (their mechanisms) to produce outcomes is shaped by contextual features. The goal of realist review is to *explain* how contextual features shape the mechanisms through which a programme works. This is achieved through testing and refining programme theories, expressed as Context-Mechanism-Outcome configurations (CMOCs). Initially, these are tentative ideas; as the review progresses and these ideas are brought into conversation with evidence (i.e. tested in relation to the evidence) they are refined to produce a more detailed explanation of how context shapes mechanisms [20, 21]. A refined theory can support the process of adapting the intervention to local circumstances.

### Summary of existing middle range theory (MRT)

Our existing realist programme theory, reported in a previous paper, put forward a number of ideas about how and why partnerships work [18]. Building on others, we incorporated Partnership Synergy Theory as a Middle Range Theory (MRT) [22, 23]. This theory posits that collaborations achieve performance benefits once an effective combination of skills, knowledge, and resources of partners (termed ‘synergy’), is achieved. Our MRT also incorporated the trust-building loop by Vangen and Huxham (2003) and its focus on risk taking as a driver of collaborative, rather than competitive, attitudes and behaviours [15]. Within the trust building loop, trust acts as an enabler for organisations to enhance their risk tolerance. Further, we identified a role for ‘faith’ [18] where individually and collectively, actors have belief that the collaborative endeavour (the intervention) is a virtuous and beneficial undertaking, thereby worthy of working on. Faith is thus likely to drive actors to dedicate time and effort to engaging in collaborative behaviour, but will change over time in response to other mechanisms and contextual factors.

As such, we posit that trust (with its link to risk tolerance) and faith serve as dual drivers for actors to begin behaving collaboratively; and they also serve as mechanisms in realist terms. According to realist theorists, mechanisms are defined as changes to people’s mindsets or behaviours introduced by the intervention [21, 24]. In our MRT, building trust and faith is the purpose and key processes that constitute the ‘collaborative functioning’ stage of collaborations. Achievement of a high level of trust and faith across the organisation allows for a synergistic state in which partners achieve maximal collaborative behaviour. Our prior work also indicated that in more integrative types of collaboration (such as a merger) or those that are mandated, trust may be progressively replaced by ‘confidence’ in contractual mechanisms as a means for driving collaborative behaviour [18]. This is because much of the risk of engaging in collaborative behaviour is enshrined in contractual obligation rather than the building of trusting, robust interpersonal relationships.

**Fig. 1:**
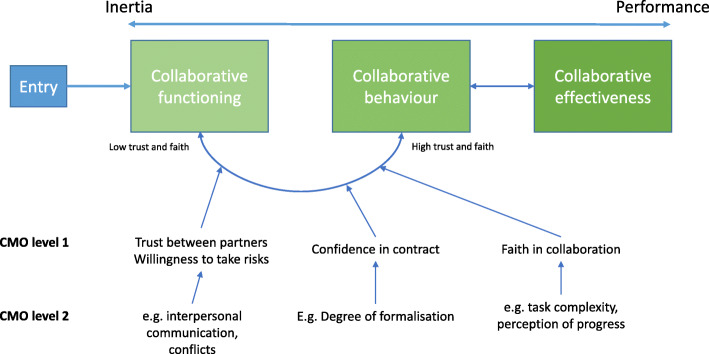
Simplified depiction of our Middle-Range Theory and the essential roles of trust, confidence, and faith. Adapted from Lasker, Weiss and Miller (2001) & Aunger et al. (2021)

Our MRT identifies that, at least initially, collaboration requires daily effort to maintain interpersonal ties and build relationships for ‘collaborative functioning’ to be maximised. Mechanisms found to be key to collaborative functioning identified in the review were trust and faith, with conflict, interpersonal communication, leadership, and cultural integration being within different CMOCs (Fig. 1) [18]. As is evident in this applied example, these CMOCs operate through one another, forming chains that are situated in temporal stages as the collaboration develops over time (Fig. 1). Our MRT posits that once trust and faith reach a certain threshold, a novel state is entered, termed “partnership synergy”, in which the benefits of collaboration can be attained. In our paper, this has been reframed as ‘collaborative behaviour’, to make ‘synergy’ a more tangible concept [18]. Driven by the integration of skills, knowledge, and resources of partners, performance benefits may include innovations brought about by sharing of expertise, cost savings from better bargaining power, and reduced duplication of effort across health systems. For this paper, we adopt our theory from phase 2 as the MRT for understanding in this phase.

### Objectives

The purpose of this paper is to present findings from the third stage of theory refinement (Fig. 1). Drawing on additional case study and organisational evaluation literature, we aim to (1) test our initial CMOCs with additional evidence and identify new CMOCs. The paper does so to (2) better understand how CMOC configurations of inter-organisational collaboration are chained together causally and how (3) differences between contexts affect their implementation. It argues that a better understanding of these elements serves to enhance usefulness of a realist theory for policy makers and practitioners tasked with developing implementing different forms of collaboration in a healthcare setting.

## Methods

### Details of search strategy

**Fig. 2:**
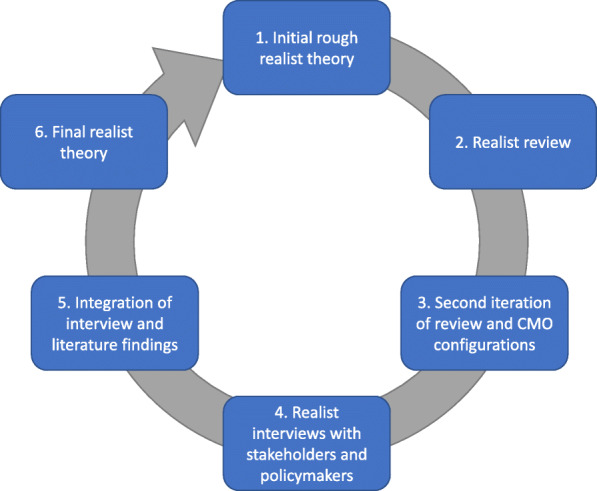
Evolution of literature synthesis by phase of review [18, 26]

The literature for this refinement stage of our realist synthesis was identified through a combination of existing literature from prior stages (systematically-searched), novel systematic searches, and grey literature sources (for identifying organisational reports and evaluations), as is typical of a realist synthesis [25]. An explanation of prior literature searches for phase 2 of the realist synthesis can be found at its site of publication [18]. Our existing searches were conducted in early 2020 on Healthcare Management Information Consortium (HMIC), MEDLINE, Social Policy and Practice, and PsycINFO databases (see additional file 1 for full search strategy). Only case studies from the existing search were included here, with these case studies being brought over into this refinement stage of the synthesis after being screened for relevance and rigour for this new phase. In addition, a novel systematic search was conducted on 10.06.20 on the Social Policy and Practice database to identify additional case studies. Further iterative searches for grey literature were conducted on 07.10.20 and 08.10.20 on UK-specific websites for evaluations of collaboration types, including the King’s Fund, the National Institute for Care Excellence, the Nuffield Trust and Health Foundation, and NHS Employers. These searches were on the publication sections of each website, with a focus on identifying evaluative reports. These used the following terms: “collaboration”, “partnership” and “integration” and were limited to 2012 onwards to maximise relevance to contemporary developments in collaborative arrangements. Figure 2 depicts the full evolution of this realist synthesis from a literature perspective.

### Inclusion criteria

We included papers that 1) were case studies or evaluations (defined as reporting results of arrangements using descriptive methods), 2) report on an inter-organisational collaboration between healthcare providing organisations, 3) and were in English language, due to resource limitations of the study. Some papers had both literature review and case study portions, and these were also included, but data extraction was performed only on the case study parts. Selected studies were then subject to rigour and relevance checks in line with realist synthesis methodology.

### Rigour and relevance screening

In line with guidance from Wong (2018), the screening for rigour was ongoing during the analysis process and aimed primarily to increase the trustworthiness of the findings [27]. For case studies and reviews, this process involved including a CMOC only when 1) supported by clear data in included studies and 2) by multiple sources [27]. For theoretical sources of evidence, only theories that had seen significant use in the literature since publication were used in the building of our MRT and CMOCs. If documents were screened out on the basis of trustworthiness, the reasons for doing so were to be recorded. However, no studies or extracts were excluded on this basis.

### Data extraction

A custom data extraction form was created which recorded the study, collaboration type, primary driver (as best deduced from the study), CMOCs which fit into prior theory, and novel CMOCs (which could be novel in context, mechanism, or outcome), which did not fit wholesale into the prior theory. This type of custom form is typical in a realist synthesis and is included in additional file 2 [28]. Additionally, we attempted to extract whether studies were reporting on externally mandated forms of partnering or voluntary forms, but it was not always possible to determine this information, unfortunately, due to inconsistent reporting of this by authors.

### Realist methods

**Fig. 3:**
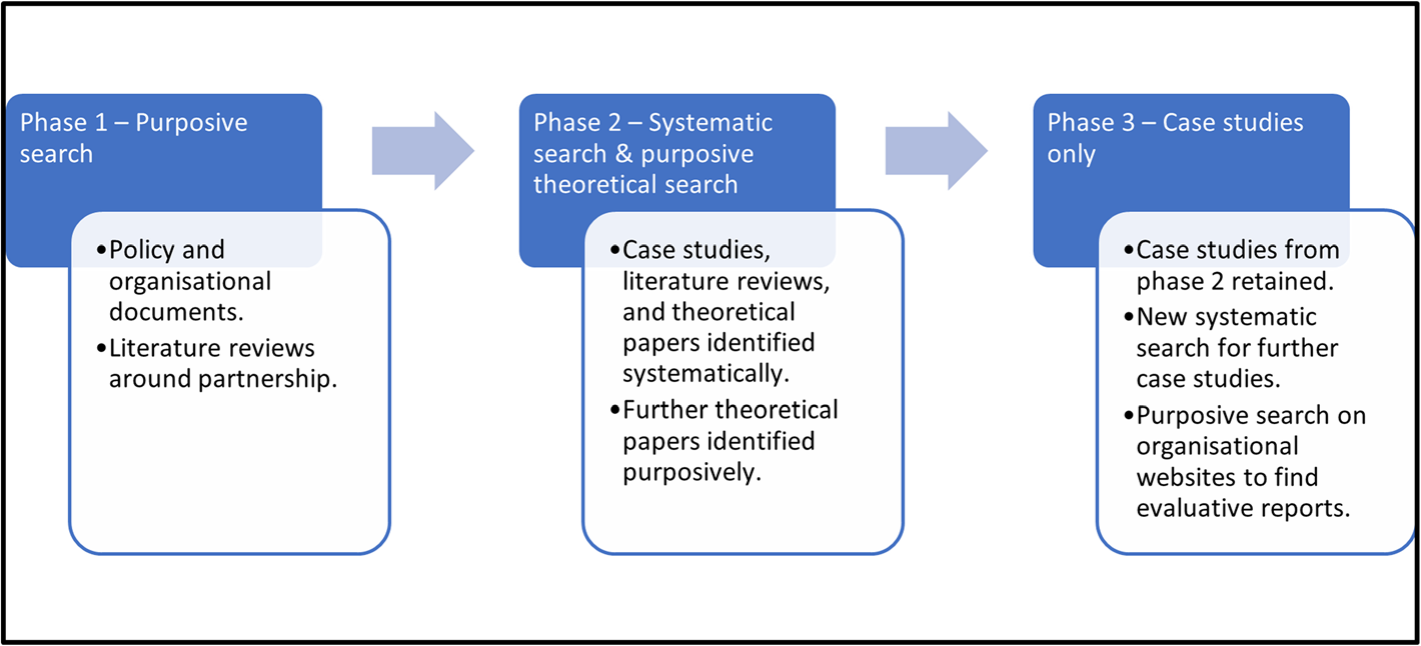
Depiction of phases and aims of this realist project, where this is phase 3 [18, 26]. Modified from Westhorp [29]

Using the existing realist programme theories from step two of this realist synthesis process as a base, we aimed to test our existing CMOCs against case studies and improve our understanding of how CMOCs are situated temporally and causally to improve our theory of collaboration in healthcare. This comprised phase 3 of our overall analysis (Fig. 3). The literature was identified through systematic searches of databases and searches of organisational websites. The literature was then categorised by collaboration type, as well as whether they were mandated or voluntary arrangements (as could best be identified), and then rigorously searched to identify CMOCs. Testing of existing CMOCs then occurred against the novel literature. This comprised identifying whether CMOCs were identical to the existing CMOCs from phase 2, or could be considered novel in terms of context, mechanism, or outcome content, or novel in terms of the relationship of one CMOC to another. Both CMOCs from the existing theory that had support, as well as novel CMOCs not present in the existing theory, were recorded. Any conflicting information about the configuration of existing CMOCs was also recorded.

This process resulted in significantly more overall CMOCs than were present in our prior realist phase, and this allowed us to gain a greater understanding of how the outcomes of certain CMOCs can become a context for another further down the chain. To identify these relationships, these CMOCs were deductively coded in NVivo 12 into categories, according to their mechanism, to better investigate the literature for presence of demi-regularities. Demi-regularities are, in realist terms, patterns of how outcomes generally come to occur [30]. These data were then used to refine the MRT and programme theories to provide a better understanding of the links between these elements. This paper was written according to the Realist And Meta-narrative Evidence Syntheses: Evolving Standards (RAMESES) II reporting standards [25].

## Results

### Literature selection

**Fig. 4:**
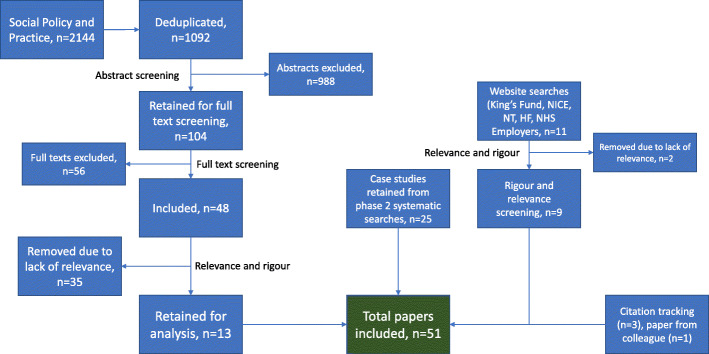
PRISMA diagram of search results

Included here after screening for relevance and rigour were 25 papers classified as case studies from searches from the second phase of our realist synthesis. For the new systematic search, the Social Policy and Practice database search identified 2144 papers, or 1092 after deduplication against our existing literature library for this project (please see Additional file1 for the full details of this search strategy). Abstracts were then screened for relevance and 104 remained. At this point, papers were most frequently excluded due to not being related to inter-organisational collaborations, or for not being case studies. After full text screening, 48 papers were considered eligible for the review, but, after screening for relevance and rigour, only 13 of these were included. All those removed were due to being insufficiently descriptive (lacking relevance). The searches on the websites of the King’s Fund, the National Institute for Care Excellence, the Nuffield Trust and Health Foundation, and NHS Employers resulted in an additional 11 papers. Nine of these were included after two were removed for lack of relevance. Three further studies were identified through citation tracking and a final paper was identified in a department newsletter after the search was completed. As a result, 51 papers were included in total in this theory refinement portion of this realist synthesis (Fig. 4) [31–81].

### Literature characteristics

Papers in the literature covered a wide range of inter-organisational collaboration types, including integrated care initiatives (such as Sustainability and Transformation Partnerships (STPs) and Accountable Care Organisations (ACOs)) (*n* = 22), mergers (*n* = 7), joint commissioning (*n* = 6), vanguards (*n* = 4), primary care networks/partnerships (*n =* 4), (*n =* buddying (*n =* 2), provider chains (*n =* 1), alliances (*n =* 1), health boards (*n =* 1), networks (*n =* 1), quality improvement collaboratives (*n =* 1) and mixed partnership types (*n =* 1). Table 1 depicts the characteristics of studies included in this review.

**Table 1:** Characteristics of included literature

Study	Country	Partnership type	Sector	Methods and sample
Adedoyin et al. *(*2016)	USA	Merger	Social work programmes	Journaling to report personal experiences and retrospective descriptions of the merger process
Allen et al. (2016)	England	Joint commissioning	Healthcare	Case study with 42 interviews & documentary analysis
Ball et al. *(*2010)	Scotland	Community health partnership/integrated care	Health and social care	More than 30 interviews with professionals, public, and voluntary sector; use of Partnership Assessment Tool [82]
Care Quality Commission (2017)	England	Sustainability Transformation Partnership/Accountable Care Organisation	Health and social care	Evaluation, based on inspection reports based on visits to 25 independent sector adult social care providers and a four-day visit to the organisation
Cereste, Doherty and Travers, (2003)	UK	Merger	Hospitals and mental health/community trusts	Focus group, and questionnaire answered by 457 trusts (mostly chief executives, finance directors, etc.)
Community Network (2020d)	England	Provider alliance/ Integrated care	Health and social care	Summary report from a wider project – case studies (methods unknown)
Community Network (2020e)	England	Provider alliance/ Integrated care	Health and social care	Summary report from a wider project – case studies (methods unknown)
Community Network (2020b)	England	Provider alliance/ Integrated care	Health and social care	Summary report from a wider project – case studies (methods unknown)
Community Network (2020a)	England	Provider alliance/ Integrated care	Health and social care	Summary report from a wider project – case studies (methods unknown)
Community Network (2020c)	England	Provider alliance/ Integrated care	Health and social care	Summary report from a wider project – case studies (methods unknown)
Cortvriend (2004)	England	Primary care trust	Acute care, primary care	Focus groups, with 31 participants taking part across five such groups, each containing 4–8 participants
Crump and Edwards (2014)	England	Provider chains	Acute care	Interviews (non-NHS, *n =* 11; and NHS, *n* = 5)
Dickinson and Glasby (2013)	England	Integrated care	Health and social care	Five case study sites, using documentary analysis, interviews, and focus groups.
Dickinson, Peck and Davidson (2007)	England	Merger	Health and social care	Case study, 23 semi-structured interviews with range of people, from service users to CEOs
Erens et al. *(*2017)	England	Integrated care	Health and social care	Case studies of 25 Integrated Care Pioneers, involving documentary analysis, qualitative interviews, and surveys
Findlay (2019)	Scotland	Health boards	Health and social care	Literature, documentary analysis, non-participant observation, and semi-structured interviews with 44 participants
Forbes, Evans and Scott (2010)	England and Scotland	Integrated care	Health and social care	Four case studies, two in England and two in Scotland; semi-structured interviews were used (*n* = 16)
Foundation Trust Network (2014)	England	Buddying	Acute care	Twelve trusts as case studies, involved in buddying; using surveys, desk research, structured interviews and documentary analysis
Fowler Davis, Hinde and Ariss (2020)	England	NHS Vanguards	Health and social care	Service evaluation with embedded team; qualitative in-depth interviews
Fulop et al. *(*2002)	England	Merger	Health and social care	Nine trusts (cross-sectional) and four trusts (case studies); using in-depth interviews and documentary analysis
Gannon-Leary, Baines and Wilson (2006)	England	Partnerships (mixed)	Health and social care and voluntary sector	Evaluation and literature review; narrative ‘experiential’ methodology
Gulliver (1999)	England	Joint commissioning, mental health	Health and social care	Evaluation; narrative ‘experiential’ methodology
Gulliver, Peck and Towell (2001)	England	Joint commissioning, mental health	Health and social care	Evaluation of a mental health service; utilising interviews with service users & staff, postal surveys, focus groups, observations, and documentary analyses
Hearld, Alexander and Shi, (2015)	USA	Alliances	Health and social care	Case study of 16 alliances; quantitative data from surveys and qualitative interviews
Henderson et al. (2020)	USA	Primary care network	Health and social care	Evaluations of six primary care clinics and community-based organisations; qualitative methods comprising 54 interviews and 10 focus groups, with review of 80 documents
Idel (2003)	Israel	Merger	Acute care	Prospective study with quantitative methods; using a questionnaire; *n* = 128 participants
Jones (2020)	England	Primary care network	Primary care	Report of experiences; narrative ‘experiential’ methodology
Kershaw et al. (2018)	England	Sustainability and Transformation Partnership	Health and social care	Case study of five STPs in London; phase 1 (small scale interviews with leaders), phase 2 (26 semi-structured interviews with leaders and stakeholders) and groups discussions.
Lalani et al.(2018)	England	Quality improvement collaborative	Acute care	Evaluation with researcher-in-residence model, based on two sites, comprising 15 semi-structured interviews
Leach et al. (2019)	England	Buddying	Health and social care	Evaluation; using quantitative performance data and mixed methods staff survey
Lewis (2005)	Australia	Primary care partnership	Primary care	Case study of two PCPs; using a network research methodology including both surveys and interviews with 37 people
Lim (2014)	UK	Merger	Health and social care	Quantitative analysis of merger data from nine hospitals relating to staff job satisfaction
Maniatopoulos et al. (2020)	UK	Vanguards (eleven different cases)	Health and social care	Comparative case studies including 66 semi-structured qualitative interviews across nine vanguards, as well as documentary analysis of included
Mervyn, Amoo and Malby (2019)	England	Network	Health and social care	Exploratory case study employing 12 initial semi-structured interviews, a literature review, and then an additional 21 interviews with another sample
Murray, D’Aunno and Lewis (2018)	USA	Accountable care organisation	Health and social care	Longitudinal case studies from 2012 to 2017 with two ACOs, including 115 semi-structured interviews and observational data based on 7 site visits
Naylor, Alderwick and Honeyman (2015)	England	Integrated care	Health and social care	Five case study sites with acute hospital providers that have moved towards integrated care, utilising 39 in-depth interviews and site visits
NHS Employers (2017)	England	Vanguards	Health and social care	In-depth case studies on three vanguards including semi-structured interviews (*n* = 13), focus groups (*n* = 3), and documentary evidence
NHS Providers (2019)	England	Integrated care	Health and social care	A briefing by a policy organisation that uses interviews (unknown number)
NHS Providers (2018)	England	Integrated care	Health and social care	Case studies from three health and social care partnerships in England, in the format of a series of organisational reports
NHS Providers and NHS Clinical Commissioners (2018)	England	Joint commissioning	Health and social care	Policy report drawing on a literature review and in-depth semi-structured interviews with clinical commissioning (*n* = 9), national thought (*n =* 5) and provider leaders (*n* = 10)
Peck, Towell and Gulliver (2001)	England	Joint commissioning	Health and social care	Case study of a combined Trust; using annual semi-structured interviews with managers, postal surveys with (*n* = 169 in 1999 and *n* = 143 in 2000), and exploratory workgroups
Pickup (2004)	England	Integrated care/joint commissioning, mental health	Adult services	Case study in the format of an ‘experiential report’
Round et al. (2018)	England	Integrated care	Primary, acute, community, mental health and social care	Programme evaluation design; using documentary analysis, 31 stakeholder semi-structured interviews, focus groups, and observational data
Shaw (2002)	England	Mergers	Health and social care, integrated Trust	Case study of merger of two trusts; using qualitative methods and semi-structured interviews with 42 people. Documentary analysis was also used
Smith et al. (2020)	England	Primary care networks	Primary care	Qualitative cross-comparative case study across four sites using: rapid evidence assessment, a workshop with academics and policy experts, interviews with stakeholders, observations, survey, documentary analysis
Southby and Gamsu (2018)	England	Integrated care, primary care networks	Primary care and voluntary and community sectors	Case study design comprising four cases, each with a GP and VCS organisation; using 18 semi-structured interviews with GPs, practice managers, practice nurses, and senior managers, and a focus group of 14 participants
Southwark and Lambeth Integrated Care (2016)	England	Integrated care	Health and social care	Report regarding organisational experience of an integrated care programme; using evaluative as well as anecdotal evidence
Starling (2018)	England	Vanguards	Health and social care	Case studies, interviewing 45 middle-to-senior clinical and non-clinical leaders and evaluators across eight vanguard sites
Steininger et al. (2016)	Austria	Hospital merger	Acute care	Qualitative case study of the merge of IT systems; involving interviews with 40 stakeholders
The King’s Fund (2005)	England	Joint commissioning	Community care	Report as part of an evaluation; observation and interviews were used (unclear quantities)
Timmins (2019)	England	Integrated care	Health and social care	Analysis of leaders’ experiences with integrated care and collaboration in a report format; based on interviews with 16 chairs and leads

### Refining the CMOCs of collaborative functioning

This refinement of our realist synthesis gave greater insight into how CMOCs are situated in the timeline of development of collaborations, which is how we have structured this section of the paper. Additionally, it has given further insight into atemporal mechanisms (those mechanisms that can activate at any time) and how these affect the process of collaborating.

### CMOC coding and establishment of CMOC chains, refinement of mechanisms

Extraction of CMOCs from included studies resulted in 338 CMOCs being identified, many of which were functionally identical and analogous to demi-regularities. The full list of these can be viewed in additional file 2. As such, there are too many CMOCs to explore here in detail, and the majority of them have been explained in our prior paper [18]. As previously mentioned, coding of CMOCs was performed according to which mechanism a context activates. The below ‘initial mechanisms’ were used as preliminary deductive codes; however, these were updated as CMOCs with other mechanisms were identified in the literature (Table 2). By the end of the process, novel CMOCs were not being identified as they were all analogous to demi-regularities that had been already found. In terms of frequency, those most identified were CMOCs with trust or confidence as the mechanism, then ‘perception of progress’, faith, interpersonal communication and information sharing, task complexity, cultural assimilation, conflict, and clarity and sharedness of vision. For the sake of brevity and due to the complexity of inter-organisational collaboration, every CMOC and the contextual factor will not be explored here, but they can be found in additional file 2 along with the full table of CMOCs identified in each respective paper.

**Table 2:** Mechanisms present in prior phase of realist synthesis (left) vs. refined theory (right), an explanation of these mechanisms, and which outcome these mechanisms typically produce

Initial mechanisms	Refined mechanisms and their type	Explanation	Most frequent outcome
Task achievement and performance	Effectiveness through collaboration: enabling innovation, reduced duplication of effort, sharing of best practices, increased access to resource, reduced gaps in services, increased influence over others	The ‘ultimate outcomes’ that usually underlie actual improvements to key metrics of organisational performance	n/a
Synergy and collaborative inertia	Changes towards collaborative behaviour from competitive behaviour (behaviour)	A move from competitive organisational behaviours to collaborative ones	Collaborative effectiveness
n/a	Risk threshold (cognitive process)	How much risk an organisation is willing to take on with a collaborator	Collaborative behaviour
Faith	Faith (cognitive process)	A belief in the collaborative endeavour as a positive force and therefore a motivation to work on its goals	Collaborative behaviour
Perception of progress	Perception of progress (mindset)	Whether actors perceive advancement towards the goals of the collaboration	Faith
Conflict	Conflict (mindset)	The perception by organisational actors that they are in opposition to collaborators in some way	Trust
*n/a*	Approach to conflict resolution and accountability (cognitive processes)	Processes and attitudes in place that lessen the severity of conflict	Conflict
Trust	Trust (cognitive process)	*“A psychological state comprising the intention to accept vulnerability based upon positive expectations of the intentions or behaviour of another*” [83].	Risk threshold
Confidence	Confidence (cognitive process)	A belief that a collaborator will behave collaboratively due to contractual or other obligation	Risk threshold
Initial trust	Initial trust (cognitive process)	Trust that manifests as a result of pre-existing contextual factors	Trust
Power	*n/a (now a contextual factor)*	Whether one organisation has more influence on proceedings than another	Trust
Leadership	*n/a (now a contextual factor)*	The set of behaviours and attitudes that key organisational leaders possess	Trust
Cultural integration	Cultural assimilation (cognitive process)	How well actors between organisations are aligning in terms of attitudes and behaviours	Trust
Interpersonal communication/ coordination	Interpersonal communication & information sharing (behaviour)	The behaviour of communicating and sharing information	Trust
Perception of task complexity	Perception of task complexity/initial faith (cognitive process)	How complex actors perceive the collaborative endeavour to be	Faith
*n/a*	Clarity and sharedness of vision (cognitive process)	How well-defined and to what extent the vision between partners is agreed-upon	Trust
*n/a*	Perceived legitimacy of collaboration (cognitive process)	How actors perceive the collaboration in terms of its authenticity	Initial faith

### Mechanisms specific to early stages of collaboration

Our initial CMOCs [18] identified that, essential to establishing ‘initial faith’ (i.e., whether engaging in collaboration is feasible, and worth the risk and effort) are factors such as financial constraints, the regulatory environment and its favourability to collaboration, and organisational size (which may affect the perceived difficulty of the task). A further contextual factor identified in this refinement stage was the reputation of the specific form of collaboration being considered; for example, some papers referred to negative perceptions due to collaborations being associated with privatisation of the NHS [67]. This lowered actors’ desire to engage with this form of collaboration in the first place (their initial faith in the endeavour). Also tied into faith as a precursor mechanism is the perceived legitimacy of collaboration, which often affects a collaboration from the outset. Impacting this mechanism is stakeholder involvement, which can serve to increase its legitimacy in the eyes of staff, whether a partnership is voluntary or not, and whether staff perceive the collaboration as a threat professionally [48].

Related to the level of initial faith is that of initial trust. Initial trust was put forward in phase 2 of our realist synthesis as likely to come into play in the ‘Connecting’ phase of collaboration, during which organisations seek partners and establish initial relationships [84]. Contextual elements identified as essential to determining this initial level of trust are the history of collaborating or competing between the organisations [36, 57, 67, 79], their organisational reputations [76], and at a later stage, the strength of legal agreements [31]. These factors have been found to enhance or undermine trust. For example, legal agreements can both act as an initial reassurance when relying on a partner, as well as undermine it, if they do not allow for attribution of collaborative behaviours to altruistic intent [41, 76]. Furthermore, this refinement stage identified also that a context of a known history of health system failures in the region can also lower initial trust [58], as evidenced by NHS Providers (2018), who put forward that “*a legacy of challenges led to a break-down in trust and dialogue and an entrenchment of organisational ‘fortress mentalities*’ [58]. As trust and risk are intricately linked, this level of initial trust is essential to setting the degree of risk an organisation is willing to take on with its partner, which can affect the aims and outcomes a partnership seeks to accomplish [42, 76].

### Middle stage of collaboration

During the ‘mid-life’ of a collaboration, a multitude of factors come into play which can help rapidly increase the level of trust, buffering against potential conflicts that may occur. Chief among these are ‘quick wins’ with a partner, which also help to increase faith [55, 76]. These small successes serve to rapidly bolster trust and can be increased further through factors such as seconding staff [38, 68], and having open, honest, stable, and empathetic leadership [58, 72]. Alongside these quick wins are longer-term battles, such as the need to ensure effective interpersonal communication between key organisational actors, managing conflict [42, 50, 52, 64], and either creating a new culture or helping build bridges between existing ones [33, 53, 79].

With respect to ensuring appropriate communication between collaborators, a number of contextual elements are key. Geographical proximity is one element that is difficult to mitigate, as a greater geographical distance between collaborators increases time spent building relationships significantly, by presenting a barrier to ease of arranging meetings and enabling informal interaction [44]. Greater geographical proximity was most often cited as improving communication, but can also be unhelpful if conflict is already occurring [47, 81]. Additionally, having a larger size and/or quantity of organisations involved can make communicating more difficult, due to the increased number of involved actors and moving parts [39]. Compatibility of IT systems [32, 79], joint staff appointments [52], and having regular collaboration-wide meetings can also work to increase trust as an outcome through the mechanism of interpersonal communication [75, 77]. When cultures are mismatched or not mutually understood, conflict can occur, which thereby reduces trust [68]. Improving cultural assimilation by configuring the context is also possible, which can also go on to enhance trust. A mutual cultural understanding can be fostered by: ensuring a shared vision of the collaboration is in place [33], by having a cross-organisational ‘inspirational leader’ who also engages in role-modelling behaviours [75], and by supporting staff through the transition [48]. It can also be improved by having joint teams of staff to work on shared goals, which can improve a sense of collegiality [46, 80]. It was evident in the literature that certain passive elements, such as the pre-existing degree of cultural distance, and whether or not the collaboration is perceived as forced upon staff, can also significantly change the difficulty of cultural assimilation [72].

### Atemporal elements impacting collaboration

Conflicts between organisations can occur as a result of deteriorations in trust, as a result of ‘acute events’ such as failures on specific tasks, or from accumulating tensions caused by cultural distance [18, 42]. Conflicts directly cause a loss of faith in the collaboration and trust between partners [18]. As such, there is a reciprocal relationship between both conflict and trust, and conflict and faith (Fig. 4). This is supported by excerpts such as: “*Conflict, for example, due to competition between partners, increases the difficulty in predicting the partner’s behaviour and increases the uncertainty in the decision to trust*” [76]. Reductions in faith can also lead to intra-organisational conflict [18]. We also found that conflict can be modulated when it occurs by the approach to conflict resolution. Conflict resolution is now a mechanism in itself in this refined theory, which can dampen the impact of conflict on trust or faith [52].

This refinement stage further identifies that use of external, impartial deal brokers and committees can lessen the impact of conflict on trust by moving the locus of that trust to the third party rather than the partner [50], along with having robust governance structures which are not imbalanced in either direction in terms of power [52]. Likewise, conflict itself can be mitigated by leaders bringing a constructive approach to conflicts, by proactively attempting to reduce power imbalances, and by avoiding or managing any senses of takeovers in the case of mergers or other more integrative collaboration types [76]. While conflict interacts with both trust and faith, so too does the degree to which the collaborative vision is shared and its clarity. The clarity of vision is more keenly interlinked with faith, which is supported by quotes such as “*most sources concur that a clear vision and/or mission statement should include attainable goals and that lack of clarity about vision can be a serious barrier to engagement*” [50]. Here, engagement can be considered similar to our concept of faith. However, the sharedness of the vision works through the mechanism of trust, as sharedness relates directly to the inter-organisational perception of each organisation. The clarity and sharedness of vision are affected by patient and public engagement (which helps keep the focus on improving care quality rather than secondary objectives), having inclusive decision-making processes, and stable leadership. In many cases, significant leadership turnover meant starting over with trust building exercises, due to large changes in vision occurring [60, 63].

As a final look into the role of faith, a key mechanism which links into faith as an outcome is ‘the perception of progress’. The perception of progress is interwoven with faith - but is not entirely the same concept (as one can have faith without much perception of progress). The perception of progress is essential for ensuring that momentum is maintained and that there is no stall into what is termed ‘collaborative inertia’, a situation in which there is insufficient faith to maximise work on the collaboration [75]. Having an increased perception of progress increases faith, and a lesser perception of forward momentum reduces it. This is supported by quotes from healthcare leaders such as the following: “*So it is harder and less dynamic at the start, until you get a drumbeat going. Then it becomes easier because the peer group start doing it for you*” [72]. Affecting this perception of progress are contextual factors such as appropriate degrees of ambition (overambition can lead to disappointment) [54, 56], implementation of ‘quick wins’, having effective planning, which ensures staff are working on the most appropriate projects at the right time [65], and, importantly, having effective evaluation and dissemination processes which ensure staff are aware of the progress being made [54, 85].

**Fig. 5:**
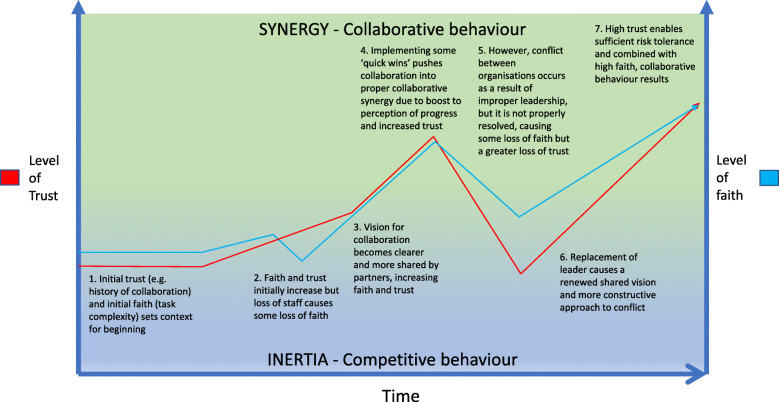
Example of how contextual elements and mechanisms may operate over time to drive a voluntary-type partnership into collaborative synergy. The lines represent how levels of trust and faith may change independently over time in response to the various numbered events

These various review findings suggest a web of contextual elements operating through many mechanisms, to produce many outcomes, forming causal chains (Fig. 5). Some of these can be altered to be more beneficial to implementers (e.g., keeping ambitions realistic), and some cannot (e.g., geographical proximity). While these elements discussed are those which underlie the functioning of the partnership, the review also identifies mechanisms which underlie material improvements to organisational performance.

### Refining CMOCs driving partnership performance

As trust and faith are maximised, work on managing conflicts, building collaborative structures, and establishing relationships, decreases. As a result, time and effort are freed up to create the conditions for collaborative ‘synergy’, which in our refined theory, refers to a willingness to engage in collaborative, rather than competitive, behaviour (Fig. 4). Once risk tolerance and faith are maximised, partners can strike out to work on risky innovations together, share best practices, pool resources, bid for contracts together, increase market power, reduce duplication of effort, and better focus on inequalities in the health system. These are the mechanisms through which ‘ultimate outcomes’ of collaboration (i.e., performance improvements) are realised - occurring as a result of an accumulation of skill, knowledge, and resources being brought to bear against problems encountered by the partnership. However, these benefits seldom come to fruition due to the sheer amount of work and goodwill that is required to rise above the quagmire of the daily functioning of the partnership, explaining why many collaborations are not successful. Studies capture contexts where “*too much was being expected too soon… as they were still working out how to function operationally before they could accomplish goals such as decreasing workload and improving care*” [79].

Achievement of this synergistic state is extremely difficult as many collaborative endeavours are ‘set up for failure’ due to initial contextual conditions being configured in such an unfavourable manner that it becomes insurmountably difficult to build the relationships required [12]. These difficult contextual conditions can include a regulatory environment that still revolves around competition rather than collaboration [52], mandated partnerships and integrated care initiatives that do not allow for building of relationships, brought into place in conditions of pre-existing histories of competition and ‘bad blood’ [58], and a lack of financial support or consideration provided for collaborative endeavours to be implemented properly [67]. As outlined by our theory, these common contextual factors serve to undermine initial trust in partners and initial faith in the process and increase task complexity to a degree that makes them very difficult to overcome. One quote from an included study of buddying arrangements in the UK reflected this clearly: *“Interviewees repeatedly said how difficult it would have been if their buddying arrangement had been imposed, and indeed those arrangements seen to be imposed by regulatory bodies appear to be have been the least successful”* [44]*.*

This information provides implications for cross-sector initiatives, such as ICS’ or ACOs taking place in the UK. In addition to many of these being mandated, cross-sector working brings additional challenges by requiring working between workforces of differing professional backgrounds. These professional differences manifest in a greater degree of cultural divide – a barrier which was referenced by many of the included case studies [42, 76]. Likewise, in cross-sector arrangements, the number of partners and size of the involved organisations is likely to be greater, which further increases the difficulty of communicating effectively and clearly, and results in much higher task complexity. These are all concerns which require great tact to mitigate.

### Refinements by collaboration type and collaborative functioning

Our initial rough realist theory suggested that partnerships can be characterised along a spectrum of integration from full integration (i.e., mergers) to more informal endeavours involving fewer people (i.e., buddying, clinical networks) [26]. Such differences between collaborative types (e.g., buddying vs. alliances) are also reflected in our findings as changes to contextual elements (i.e., whether they are mandated or not). These can affect the task complexity, perceived legitimacy, faith, and initial trust. Our review identifies how it is possible to trace how these impact on implementation. For example, a voluntary buddying arrangement is likely to be relatively simple to implement, as it is unlikely to be perceived as threatening by staff, does not involve many organisations, and, while perhaps not supported by formal legal agreements, is likely to involve partner self-selection. This arrangement is likely to start with a high degree of initial trust between partners and initial faith in the process.

On the other hand, ICS’ - which have now come into force in April 2021 - involve a significant number of large organisations coming together in a cross-sector manner, including local councils, primary and social care, and acute care [1]. This drastically increases task complexity by being cross-sector and having many involved organisations. Additional challenges include that it is likely such a move may be perceived as a threat by staff, reducing faith by reducing its legitimacy in their eyes. It is also likely to increase difficulty of effective communication by having both great breadth and depth of organisations involved, making trust building more difficult, and there may be reduced initial trust from outset by being set in a local context of pre-existing competitive attitudes. In addition to that, being given a rapid timeline while having to overcome prior differences and conflicts poses an additional challenge which requires a strict patient-centred focus shared across the system to overcome.

Our programme theory suggests that formalisation through contracts may be one means of enabling collaborative behaviour in such a situation where initial trust is likely to be low or complexity very high. The following section explores further how *trust* and its relationship to risk threshold may be replaced by *confidence* as a primary driver for collaborative behaviour in mandated or integrative collaboration types.

### Trust versus confidence in integrative and mandated partnership types

In phase 2 of our realist synthesis, we identified a common CMOC which suggested that the formalisation of an arrangement through contracting facilitated trust relationships by having potential to act as a buffer where trust may otherwise be lacking [18]. Inversely, trust may also be undermined if too much collaborative behaviour is mandated through contract, as they assume the partner will act collaboratively as a result of contractual obligation. As such, we also found evidence that with either mandated or otherwise more integrative types of partnerships (e.g. mergers), trust may not be the primary determinant of collaborative behaviour. That role would instead shift to confidence [18].

**Fig. 6:**
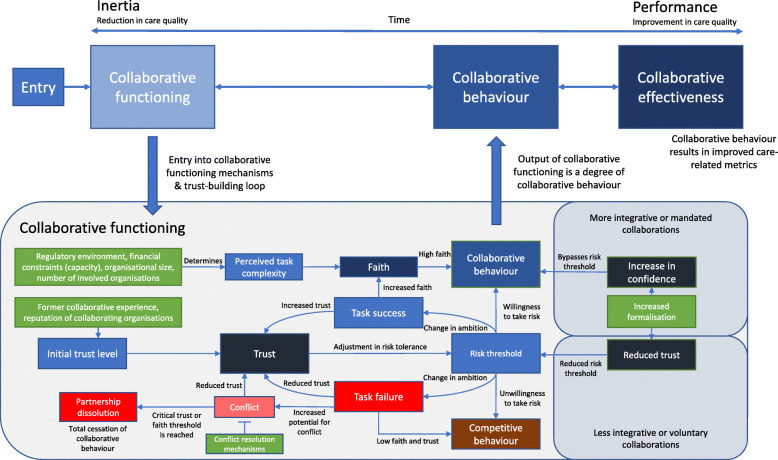
Refined programme theory diagram based on new and refined CMOCs and demi-regularities identified in this phase of the synthesis. Contextual factors are represented by green boxes

Our updated review sought to further understand the relationship between confidence, formalisation, risk tolerance, and trust. While difficult to identify relevant information, our included literature identifies an inherent mistrust taking place in organisations involved in mandated partnerships, with perceptions of being ‘taken over’, atmospheres of ‘them and us’, and domination of powerful partners resulting in a lack of trust [43]. Next to this lack of trust in mandated collaborations, formalisation was seen by sources as a means for risk management. Use of controls and contracts *“to minimise uncertainties of behaviour by partners”* had value in instances where trust was low [76]*.* Another source put forward the notion that formalisation through contract is “*primarily about managing risk, trying to situate the risk with the organisation/s most able to mitigate it, and giving them the power to do so.”* [58]*.* As such, we have added the concept of confidence, built through formalisation, to our programme theory diagram, as a determinant of risk tolerance (Fig. 6). Greater confidence (context) will thereby increase risk tolerance (mechanism) for engaging in collaborative behaviour (outcome) (Fig. 6).

However, while some sources were espousing formalisation as a means for improving trust, it was evident that other sources were warning that over-formalisation could undermine the trust-building process, as “*[the] contribution [of contracts] is less in subsequent phases once trust grows, not least because early contracts cannot anticipate every eventuality”* [76]*.* This sentiment is echoed by quotes such that “*although a lot of the joint commissioning processes described to us were formalised and structural, people often recognised that joint working is essentially relational (based on informal conversations and interactions)*” [41]. These findings indicate that voluntary collaborations should be careful not to overly rely on structural means of obligating collaborative behaviour where they should be focused on building relationships. Memoranda of understanding (MoUs), while generally non-binding, increase trust between collaborators and thereby reduce perception of risk taken on when engaging in collaborative behaviour [62]. We hypothesise that these non-binding contracts such as MoUs may be most appropriate as a tool to increase risk tolerance for less integrative or voluntary arrangements, where there is a risk of misattribution to obligation rather than genuine collaboration with the use of binding contracts [6]. As Casey (2008, p. 77) puts forward “*there is a need for a balance between power sharing and control, between processes and results, between continuity and change and between interpersonal trust and formalized procedures.”*

### A novel means of depicting CMOC chains: ‘causal webs’

**Fig. 7:**
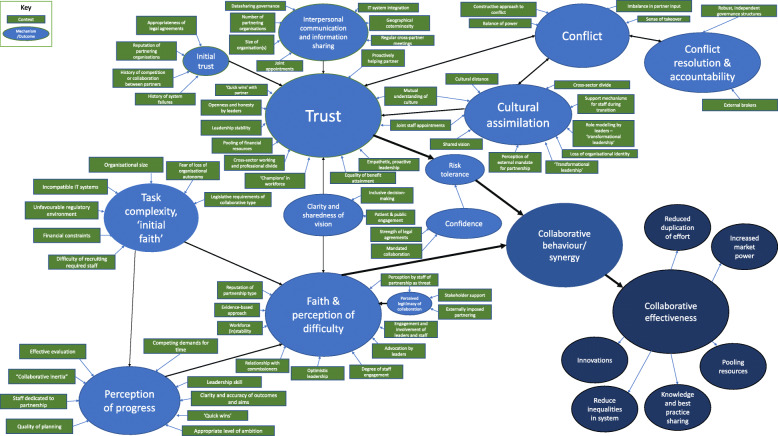
Depiction of ‘web of causality’ formed by CMOC chains of how and why healthcare collaborations work. Green and blue elements relate to Collaborative Functioning

Our review findings suggest that CMOCs formed chains of generative causality as a result of the Outcome of one CMOC becoming the Context for another. For example, a larger organisational size (context) leads to greater task complexity (mechanism), which affects people’s faith in the process (outcome) [32]. However, further down the chain, faith is a context in which collaborative behaviour (mechanism) occurs, leading to improved synergy (outcome). As such, the ‘chains of causality’ emerged naturally from the linkages between CMOCs. As there is no common diagrammatical standard for how to depict CMOC chains in realist syntheses, we found that it was appropriate to depict our findings in the ‘causal web’ shown in Fig. 7. Elements that only serve as contexts are represented in green boxes, and mechanisms/outcomes as blue ellipses. As such, in the figure, it should be simple to follow the aforementioned example of CMOC chain in the figure from organisational size (context 1) through to collaborative behaviour (outcome 2). This mode of representation draws similarities with the concept of a ‘context map’ put forward by Renger et al. (2015) for use with realistic evaluations, however, our approach expands it by including both mechanism and outcome in the web with their own means of representation. Yet, it is important to note that the diagram does not depict how specific contexts alter the mechanisms, only which mechanisms are attached to which contextual factors, and which outcomes to which mechanisms [86]. For specific dynamics, Fig. 6, updated from our version in phase 2 based on the present refinements, depicts the key mechanisms and dynamics underlying the *how of* the workings of the CMOCs we identified.

## Discussion

This phase of realist synthesis served to refine CMOCs identified in our previous paper to ensure these CMOCs are supported by case study evidence, while gaining further understanding of the temporal and causal links between them. Key refinements to the theory have included an enhanced understanding of ‘partnership synergy’ as changes towards collaborative, from competitive, behaviour, the formation of ‘initial faith’, which firmly situates multiple CMOCs into the early stages of collaborations, as well as ‘approach to conflict resolution’ becoming a mechanism that has a greater number of contextual factors changing its configuration (Fig. 7). The degrees to which the vision for the collaboration is shared and/or clear have also been added as mechanisms, due to having substantially more evidence in included literature supporting its construction as a mental process occurring within minds of actors, with its own contextual elements affecting their configuration. Next to this, the ‘perceived legitimacy’ of the arrangement has been added, due to new CMOCs being identified, which is linked into faith (Table 2). Our refinements have allowed us to make modifications to our original programme theory diagram, which was part of phase 2, depicted here in Fig. 6.

Attempting to simultaneously capture the various dynamics of inter organisational collaboration within one overarching theory is an enormously complex task. Indeed, one example alone cited Primary Care Networks as having 47 different potential organisational forms [79]. Nonetheless, these collaborative entities have more commonalities than differences when it comes to what influences their functioning. While we attempted to manage the process of analysing these collaborative types [18, 26], the approach has limitations in terms of the analytical depth that can be achieved, as a certain level of abstraction is required to maintain applicability across collaborative types. Although a realist approach has not been applied to all healthcare collaborations, others have successfully applied it to subtypes of collaboration [17, 19, 87].

Our findings build on others in suggesting that trust is closely linked with the concept of collaborative synergy (framed here as ‘collaborative behaviour’) and mutual successes [23]. Jagosh et al. (2012) put forward the idea that synergy can be both a context and an outcome, and through those means, synergy can act as a buffer against negative events [87]. In contrast to this proposition, in our refined theory we argue that synergy is instead the production of collaborative behaviour – a mechanism introduced by the intervention which exists in actors’ minds in a summative manner across the involved organisations. Synergy itself thereby cannot act as a buffer against ‘conflicts’, etc., rather, trust and faith themselves are then the elements affected by acute events such as conflict. Jagosh et al. (2015) also refer to a pre-existence of trust or mistrust, determined by community history, which set the context for their intervention, as is also reflected in the present theory [23]. However, we also build upon such examples by introducing the relationship of trust with risk tolerance, which explains *how* trust is a necessary factor for engaging in collaborative behaviour.

Also linked to risk tolerance was the concept of confidence, which came more into play when considering integrative or mandated forms of collaboration. Other sources not included in our systematic search indicate that when contracts are in place to uphold risk-sharing, benevolent actions are not attributed to free actions by the partner, but rather to the mere existence of the contract [88]. This suggests that non-binding agreements, such as memoranda of understanding commonly used in the NHS, may be more appropriate for building that relational trust [88]. The notion that more integrative or mandated partnership types may require a more contractual governance structure, whereas less integrative or voluntary partnerships may rely on a more relational structure, is supported by other findings in the literature [89]. This is exemplified by the finding that “*the strongest effect measured in the model is the overall effect of relational norms on project performance (including the mediating effect of partners’ trust and partners’ contribution)”* [89]. Likewise, they identified that project complexity affects the uncertainty about the partnership project, requiring greater structural and relational governance to properly overcome. This understanding underlies our programme theory, which suggests that in more integrative or mandated collaborations, formalisation through contracts reduces risk to engage in collaborative behaviour by making the collaborative behaviour obligatory. However, in more voluntary or less integrative types of collaboration, having binding formalisation can lead to misattribution of altruistic behaviour to the existence of the contract, undermining trust.

This evidence suggests that the need for formalisation to underline the mechanism of confidence, by obligating a degree of collaborative behaviour, could be undermined by the current lack of statutory authority of such arrangements in the UK. However, since the current successful examples (e.g. ICS’) tend to be those that have evolved from pre-existing arrangements with effective leaders in place and a relatively strong history of collaboration, the addition of legislation which reduces flexibility may undermine existing ways that current ICS’ have developed [72]*.* Both of these dimensions raise a number of implications regarding the notion of trust vs. confidence (or control) and their relation to risk (e.g. [90]), indicating that these similar mechanisms are at play in inter-organisational healthcare collaborations. Das and Teng (2001) identify that both trust and control are two separate routes to risk reduction in alliances, and they also situate alliance types such as joint ventures as oriented more towards the locus of control than in non-equity alliances, which echoes our findings.

Our introduction of the concept of faith, while relatively intuitive, is also one underrepresented in existing theories of inter organisational collaboration [76]. Our findings indicate that environmental factors which are out of one’s control tend to reduce faith by presenting insurmountable obstacles, but facilitators which are in one’s control may be more closely linked to building trust. In this sense, faith can be likened to a constant cost/benefit analysis occurring in actors’ minds regarding their participation in the collaborative endeavour. Rycroft-Malone et al. (2016) have conducted a prior realist evaluation (rather than synthesis) of a type of organisational collaboration in healthcare in the UK and argued that ‘motivation to engage’ is essential for the collaborative process – a concept similar to ‘faith’ here. This motivation to engage was also linked to perceptions by stakeholders, and concepts of to whom the collaboration belongs – represented in the present theory as ‘perception of legitimacy’. These similar reflections of trust and faith in the literature add support for our realist theory of healthcare collaborations. However, we build upon these links and prior attempts at building CMOC chains by proposing what we are terming a ‘web of causality’ (Fig. 7). Although not explicitly using Partnership Synergy Theory as an MRT, Rycroft-Malone et al. (2016) put forward the notion that “*where structures, positions and resources were aligned, this released the potential and unlocked barriers for purposeful collective action*”. This corresponds strongly to our MRT and the notion of partnership functioning mechanisms being essential precursors to collaborative behaviour and the accomplishment of synergistic effects.

Our resultant framework bears similarities to others in other fields where collaboration is at its core, such as teamworking. One such example is a framework developed by Reeves & Scott (2010) for interprofessional teamworking. Teamworking is likely to constitute a subset of dynamics occurring within interorganisational collaborations [91]. This framework, particularly in the “team processes” aspect, includes similar concepts such as trust and respect, communications, conflict, and “willingness” (which relates to our mechanism of ‘faith). These theoretical similarities have also been further highlighted by a systematic review on the topic of interprofessional vs. inter-organisational collaborations in healthcare, which draws attention to how theoretical frameworks for both have been used interchangeably in the literature, while also highlighting the additional difficulties and complexity when applying such concepts across organisational boundaries [92]. As such, due to our tackling of a wide range of mixed types of healthcare collaborations, with minor modifications, we see great potential for this theory to be applied in other areas, including inter-organisational collaboration outside of healthcare settings. Our next step will be to adopt this realist theory as a framework against which we will test primary data from interviews with people involved in healthcare collaborations, to further refine and improve our understanding of this phenomenon. Additionally, we will seek to improve our understanding of how collaborative behaviour is linked to improved organisational performance.

### Limitations

Although every effort was made to be as exhaustive with the literature search as possible, including multiple waves of systematic searching throughout the process, as well as searching grey literature sources such as organisational websites, it is nonetheless possible we missed out literature that would have been informative to the present review. A particular challenge was covering all of the partnership types due to the myriad overlapping terms used to refer to various collaboration types. Additionally, while some included papers did talk about collaborations which ended in dissolution (e.g. Murray, D’Aunno and Lewis (2018) [42]), these were nonetheless underrepresented here, perhaps due to underreporting of failed collaborations arising from publication bias. Inclusion of more examples of unsuccessful collaborations would have provided further evidence regarding how the context can impact activation of mechanisms in a potentially negative manner.

One could also criticise the review for being UK-centric, however, we did include several studies from outside the UK, such as Austria [51] and the United States [47] and did not purposely remove any based on country-specific metrics. Indeed, additional information from other regulatory contexts would have only been more informative. Nonetheless, due to the scope of our project, it is likely we missed literature from other countries.

Lastly, depending on the level of analysis that one seeks to operate upon, it is possible to criticise the present theory for not delving deep enough. As previously mentioned, we had to stay at a relatively elevated level of abstraction in order to be able to encompass multiple types of inter-organisational collaboration. One could say that there are still changes to participant reasoning (i.e., mechanisms) occurring within some of the arrows present in Fig. 7. For example, organisational size (context) leads to a change in perception of task complexity (mechanism) and thereby faith (outcome). However, one could still ask *how* does organisational size lead to a change in perception of task complexity? We could postulate that it is likely due to an ongoing cognitive appraisal within actors’ minds of the workload required, and that by having a greater number of other actors involved in the collaboration they thereby gain the knowledge that complexity has increased. However, we had to keep this review process manageable and, in many cases, we did not have evidence for many aspects at this level of ontological depth. As such, we have set out some signposts of where to explore, but this remains an aspect to delve into in further research.

## Conclusion

While many existing theories have delved into *what* underlies the process of inter-organisational collaborations in healthcare, this paper builds upon our knowledge of not only *what,* but also *how* and *why* these elements work. This was achieved by performing in-depth extraction and qualitative coding of CMOCs present in 51 case studies and evaluative reports, and by forming hypotheses to produce a refined realist theory. This theory has cemented the interrelated roles of trust and risk tolerance, faith, task complexity, interpersonal communication, cultural integration, and synergistic effects with novel mechanisms, including 'perception of progress'. It also builds upon similar existing findings in the literature from other authors by linking contexts, mechanisms, and outcomes together. This allows an understanding of how initial environmental and interorganisational conditions set levels of trust, faith, and task complexity, and how these mechanisms can be managed later in the process of collaborating. Likewise, in mandated or highly integrative collaborations, the locus may be shifted from trust towards contractual obligation and a sense of confidence that the partner will act collaboratively. These chains of CMOCs were situated within a ‘web of causality’ which allowed us to depict how distant contextual items and their mechanisms work to affect more distal outcomes, including collaborative behaviour. We hope these theoretical advances inspire further empirical study and can prove useful to those seeking to establish collaborative arrangements between health organisations.

## Supplementary Information

**Additional file 1.**

**Additional file 2.**

**Additional file 3.**

## Data Availability

The datasets generated and analysed during this study are available from the corresponding author on reasonable request.

## Abbreviations

CMOC: Context-mechanism-outcome configuration
HMIC: Healthcare Management Information Consortium
NHS: National Health Service
NIHR: National Institute for Health Research
UK: United Kingdom
MRT: Middle Range Theory
PRISMA: Preferred Reporting Items for Systematic Reviews and Meta-Analyses
PROSPERO: International prospective register of systematic reviews
STP: Sustainability and Transformation Partnership
