# Supplementary material for: When trust, confidence, and faith collide: refining a realist theory of how and why inter-organisational collaborations in healthcare work
Source: BMC Health Serv Res. 2021 Jun 26;21:602. doi: 10.1186/s12913-021-06630-x (PMC8235919; doi:10.1186/s12913-021-06630-x)
Supplement: Supplementary file 1 — Additional file 1. [file 12913_2021_6630_MOESM1_ESM.docx]

# Inclusion Criteria

Criteria for inclusion:

1. Paper clearly relates to collaborations between one or more organisations on either a structural or individual level.
2. Paper is a case study or review that contains case studies.

Criteria for exclusion:

1. Relates to collaborations or partnerships between staff and patients rather than between organisations (intraorganisational).

Results as of 10.06.2020: 2144

After deduplication: 1092

Databases: Social Policy and practice

Search strategy

1

partnering.mp. [mp=abstract, title, publication type, heading word, accession number]

2

partnership.mp. [mp=abstract, title, publication type, heading word, accession number]

3

"joint working".mp. [mp=abstract, title, publication type, heading word, accession number]

4

merger.mp. [mp=abstract, title, publication type, heading word, accession number]

5

acquisition.mp. [mp=abstract, title, publication type, heading word, accession number]

6

alliance?.mp. [mp=abstract, title, publication type, heading word, accession number]

7

"partnership working".mp. [mp=abstract, title, publication type, heading word, accession number]

8

buddying.mp. [mp=abstract, title, publication type, heading word, accession number]

9

(clinical adj1 network).mp. [mp=abstract, title, publication type, heading word, accession number]

10

(coordinating or co-ordinating or coordination or co-ordination).mp. [mp=abstract, title, publication type, heading word, accession number]

11

"joint commissioning".mp. [mp=abstract, title, publication type, heading word, accession number]

12

vanguard.mp. [mp=abstract, title, publication type, heading word, accession number]

13

healthcare.mp. [mp=abstract, title, publication type, heading word, accession number]

14

hospital?.mp. [mp=abstract, title, publication type, heading word, accession number]

15

public health.mp. [mp=abstract, title, publication type, heading word, accession number]

16

mental health.mp. [mp=abstract, title, publication type, heading word, accession number]

17

primary care.mp. [mp=abstract, title, publication type, heading word, accession number]

18

(case adj1 (study or studies)).mp. [mp=abstract, title, publication type, heading word, accession number]

19

1 or 2 or 3 or 4 or 5 or 6 or 7 or 8 or 9 or 10 or 11 or 12

20

13 or 14 or 15 or 16 or 17

21

evaluation.mp. [mp=abstract, title, publication type, heading word, accession number]

22

18 or 21

23

19 and 20 and 22
