# Supplementary material for: When trust, confidence, and faith collide: refining a realist theory of how and why inter-organisational collaborations in healthcare work
Source: BMC Health Serv Res. 2021 Jun 26;21:602. doi: 10.1186/s12913-021-06630-x (PMC8235919; doi:10.1186/s12913-021-06630-x)
Supplement: Supplementary file 2 — Additional file 2. [file 12913_2021_6630_MOESM2_ESM.docx]

Included as CMO if any context is mentioned

‘Barriers’ may be out of org. control and lower faith, whereas facilitators which can be actioned are more linked to trust

| Study | Partnership type | Key driver | Existing CMOs | New CMOs | Mandated/voluntary (where clear) |
| --- | --- | --- | --- | --- | --- |
| (Adedoyin et al., 2016) | Merger | Efficiency | - Conducive environment for discussion 🡪 more interpersonal communication 🡪 certainty/faith - Ongoing ‘external’ evaluation 🡪 novel unbiased perspective 🡪 conflict reduction | - Organizing clusters of personnel from both organisations 🡪 cultural commonality 🡪 reduced conflict - Seek understanding of differing cultures 🡪 reflection on existing culture 🡪 shared vision - Mutual understanding of culture 🡪 trust 🡪 shared vision - Honest dialogue 🡪 improved trust 🡪 reduced conflict - Shared vision 🡪 improved faith 🡪 task accomplishment |  |
| (Allen et al., 2016) | Joint commissioning | Efficiency | - History of competition with an organisation 🡪 reduced trust 🡪 increased conflict | - Regulatory uncertainty between competition and collaboration 🡪 slowed task accomplishment 🡪 reduced faith - Regulatory freedom to collaborate 🡪 reduced competitive behaviour 🡪 increased faith - Reduced competitive bidding 🡪 reduced workload 🡪 improved task accomplishment - Mandated collaboration 🡪 lack of faith 🡪 reduced synergy | Mandated |
| (Ball et al., 2010) | Community health partnership/integrated care | Care quality | - High enough trust 🡪 risk management - Effective communication 🡪 improved trust 🡪 reduced conflict - Background of effective collaboration 🡪 improved trust 🡪 improved task accomplishment | - Development of enabling environment 🡪 better trust 🡪 reduced conflict - Alignment and pooling of budgets 🡪 greater trust 🡪 reduced conflict - ‘Inclusive ethos’ 🡪 increased trust 🡪 reduced conflict | Mandated |
| (Care Quality Commission, 2017) | Sustainability Transformation Partnership/Accountable Care Organisation | Care quality | - Prior poor relationships between orgs. 🡪 reduced trust 🡪 increased conflict - Lack of shared vision 🡪 reduced trust 🡪 increased conflict - Collaborative inertia 🡪 Lack of task accomplishment 🡪 collaborative inertia | - Workforce churn 🡪 lack of task accomplishment 🡪 lack of faith - Lack of staff engagement in process 🡪 lack of faith 🡪 conflict - Leadership turnover 🡪 inability to build trust 🡪 increased conflict - Lack of IT system integration 🡪 reduced information sharing 🡪 reduced task accomplishment |  |
| (Cereste et al., 2003) | Merger | Efficiency |  | - Greater stakeholder support 🡪 increased faith 🡪 reduced conflict | Mandated |
| (Community Network, 2020d) | Provider alliance/ Integrated care | Care quality | - Shared vision 🡪 increased trust 🡪 reduced conflict | - Empathetic leadership 🡪 improved trust 🡪 reduced conflict - Starting with small problems 🡪 improved task accomplishment 🡪 increased trust - Involving staff in process 🡪 reduced workforce churn 🡪 improved task accomplishment |  |
| (Community Network, 2020e) |  |  | - Background of effective collaboration 🡪 improved trust 🡪 improved task accomplishment - Shared vision 🡪 increased trust 🡪 reduced conflict | - IT system integration 🡪 improved information sharing 🡪 task accomplishment |  |
| (Community Network, 2020b) |  |  |  | - Staff in position dedicated to service transformation 🡪 greater task accomplishment (for partnering) 🡪 reduced inertia - Leaders espousing virtues of collaboration 🡪 increased faith 🡪 reduced conflict |  |
| (Community Network, 2020a) |  |  |  | - Shared accountability 🡪 improved trust 🡪 reduced conflict - Strong shared vision by leadership 🡪 improved cultural integration 🡪 improved trust - Joint meetings and co-locating staff 🡪 improved cultural integration 🡪 improved trust - Support mechanisms for staff during change period 🡪 improved cultural integration 🡪 improved trust |  |
| (Community Network, 2020c) |  |  | - Geographical coterminosity 🡪 greater communication 🡪 increased trust - Shared vision 🡪 increased trust 🡪 reduced conflict |  |  |
| (Crump & Edwards, 2014) | Provider chains | Efficiency | - Ongoing evaluation 🡪improved task accomplishment 🡪 improved trust - Geographical coterminosity 🡪 greater communication 🡪 increased trust - Greater geographical distance 🡪 reduced task accomplishment 🡪 reduced trust | - Multisite providers require single management system (e.g. central head office) 🡪 reduction in task complexity 🡪improved synergy - Site manager leadership maintains values of chain leadership 🡪 maintenance of shared vision 🡪 improved trust - ‘Talent management’ and enhancement 🡪 improved task accomplishment 🡪 improved trust - Espousal of ‘good followership’ 🡪 improved culture 🡪 improved trust - Innovation 🡪 processes for dispersing innovative practice 🡪 synergy - Rolemodelling by leaders 🡪 improved culture 🡪 improved trust - Identification of unstated aims 🡪 improved task achievement 🡪 improved trust - High quality planning/due diligence 🡪 improved task achievement 🡪 improved trust |  |
| (Dickinson & Glasby, 2013) | Integrated care | Care quality | - Geographical coterminosity 🡪 greater communication 🡪 increased trust - Involvement of stakeholders (service users and carers) 🡪 increased authenticity of arrangement 🡪 increased faith | - Alignment and pooling of budgets 🡪 greater trust 🡪 reduced conflict - Operationalisation of pooled budget 🡪 innovation 🡪 improved task achievement |  |
| (Dickinson et al., 2007) | Merger | Efficiency |  | - ‘Transformational leadership’ 🡪 improved cultural assimilation 🡪 improved trust & faith - Perception of merger by staff (i.e. not as a takeover) 🡪 cultural assimilation 🡪 trust & faith - Joint meetings and co-locating staff 🡪 improved cultural integration 🡪 improved trust - Too much consensus 🡪 collaborative inertia 🡪 reduced task achievement (innovation) - Having too many partners (breadth) 🡪 reduced interpersonal communication (depth) 🡪 reduced trust | Mandated |
| (Erens et al., 2017) | Integrated care | Care quality | - Increased organisational size 🡪 increased task complexity 🡪 reduced task accomplishment - Compatible IT systems 🡪 reduced task complexity 🡪 greater task accomplishment - Cultural closeness 🡪 reduced conflict 🡪 improved trust | - Strong commitment 🡪 improved faith 🡪 synergy - Information governance making data sharing difficult 🡪 reduced task accomplishment 🡪 reduced faith - Conflicting government policy 🡪 increased task complexity 🡪 reduced faith - Competing demands for time 🡪 reduced task accomplishment 🡪 reduced trust, collaborative inertia |  |
| (Findlay, 2019) | Health boards | Care quality | - Shared values and purpose 🡪 improved trust 🡪 reduced conflict - Strong interpersonal communications 🡪 improved trust 🡪 reduced conflict | - Strong commitment to partnership as a form of governance 🡪 increased faith 🡪 reduced conflict - Strong history of successful partnership working 🡪 increased faith 🡪 reduced conflict - Lack of clarity 🡪 lack of faith 🡪 lack of task accomplishment - Absence of key players 🡪 lack of leadership 🡪 lack of authority - Poor behavioural practices 🡪 reduced faith 🡪 reduced engagement - Passivity rather than genuine engagement 🡪 lack of faith – reduced engagement - Strong linkages across tiers of organisation 🡪 greater faith 🡪 greater task accomplishment - Partnership working becomes habitual 🡪 synergy 🡪 effectiveness - Open and honest relationships between senior actors 🡪 trust 🡪 synergy - Leaders being advocates for partnership 🡪 increased faith 🡪 reduced conflict - Leaders being role models (behaviourally) 🡪 enhanced cultural uptake 🡪 increased trust - Constructive approach to conflict by leaders 🡪 improved conflict resolution 🡪 reduced conflict - Uncertainty about parameters of partnership working 🡪 reduced task accomplishment 🡪 reduced faith - Robust structures independent of individual partner characteristics 🡪 improved conflict resolution 🡪 improved trust - Significant time commitment for partnership functioning 🡪 temporarily reduced organisational performance 🡪 reduced faith |  |
| (Forbes et al., 2010) | Integrated care | Care quality | - Previous experience of joint working 🡪 initial trust level 🡪 initial risk appetite | - Cross-sector working 🡪 lack of inter-sector trust for managing services 🡪 increased conflict - Imbalance of partner input 🡪 increased conflict 🡪 reduced trust - Conflicting priorities 🡪 conflict 🡪 reduced trust - Sense of ‘take over’ 🡪 increased conflict 🡪 reduced trust - Administrative burden of systems integration 🡪 reduced task accomplishment 🡪 reduced faith/trust - Improper evaluation (wrong performance metrics) 🡪 perceived lack of success 🡪 reduced faith - Financial deficit 🡪 increased task complexity 🡪 reduced faith - Lack of policy guidance 🡪 increased task complexity 🡪 reduced faith - Increased workload 🡪 increased conflict 🡪 reduction in faith | Mandated |
| (Foundation Trust Network, 2014) | Buddying | Care quality | - Shared values 🡪 improved trust 🡪 reduced conflict - Greater geographic distance 🡪 reduced communication 🡪 reduced trust - Cultural closeness (context) 🡪 reduce conflict (mechanism) 🡪 avoiding degradation in trust (outcome | - Increase in workload of core personnel 🡪 reduction in task accomplishment 🡪 reduction in faith - Access to external expertise 🡪 innovation 🡪 synergy - Organisational freedom to choose partners 🡪 improved trust 🡪 reduced conflict | Mandated & voluntary |
| (Fowler Davis et al., 2020) | Vanguards | Care quality |  | - Inadequate funding 🡪 reduced task accomplishment 🡪 reduced faith & trust - Unclear outcome metrics 🡪 reduced perception of success 🡪 reduced trust & faith |  |
| (Fulop et al., 2002) | Merger | Efficiency | - Greater geographic distance 🡪 reduced communication 🡪 reduced trust - Workforce churn 🡪 reduced task accomplishment 🡪 reduced trust | - Collaborative inertia 🡪 reduced task accomplishment 🡪 reduction in care quality - Pulling together of clinicians 🡪 share of good practice 🡪 synergy - Larger trust size 🡪 more sharing of ideas 🡪 synergy - Larger trust size 🡪 more remote managers 🡪 loss of informality/ interpersonal communication - Greater cultural differences 🡪 greater task complexity 🡪 reduced task achievement |  |
| (Gannon-Leary et al., 2006) | Partnerships (mixed) | Care quality |  | - Unclear outcomes 🡪 unclear task achievement 🡪 reduced task accomplishment - Increase in workload of core personnel 🡪 reduction in task accomplishment 🡪 reduction in faith - Fear of loss of organisational autonomy 🡪 reduced faith 🡪 increased conflict - Information governance making data sharing difficult 🡪 reduced task accomplishment 🡪 reduced faith - Access to external brokers 🡪 avoidance of conflict 🡪 increased trust - Participatory and democratic governance arrangements 🡪 increased trust 🡪 reduced conflict |  |
| (Gulliver, 1999) | Joint commissioning, mental health | Care quality | - Shared vision 🡪 increases trust 🡪 reduction in conflict - Workforce turnover 🡪 reduced trust 🡪 increased conflict | - Commitment by leadership 🡪 improved faith 🡪 reduced conflict |  |
| (Gulliver et al., 2001) | Joint commissioning, Mental health | Care quality |  | - Loss of organisational identity 🡪 increased difficulty of cultural merger 🡪 increased task complexity - Co-location of staff 🡪 greater interpersonal communication 🡪 greater cultural assimilation - Lack of shared vision 🡪 loss of staff autonomy 🡪 reduced faith - Reduced faith 🡪 staff turnover & emotional exhaustion 🡪 reduced organisational performance |  |
| (Hearld et al., 2015) | Alliances | Efficiency |  | - Clear vision 🡪 reduction in participation costs 🡪 increased faith - Leadership perceived as effective 🡪 reduction in participation costs 🡪 increased faith & trust - Changes in leadership 🡪 reduction in clarity of vision 🡪 reduced faith - Negatively perceived leadership 🡪 change in leadership 🡪 increased faith | Voluntary |
| (Henderson et al., 2020) | Primary care networks | Care quality | - Staff turnover 🡪 reduced trust 🡪 increased conflict - Geographical coterminosity 🡪 increased inter-personal communication 🡪 increased trust | - Information governance making data sharing difficult 🡪 reduced task accomplishment - Lack of faith 🡪 prioritisation of non-partnership work 🡪 lack of task accomplishment - Hiring of dedicated partnership ‘champions’ 🡪 improved task accomplishment 🡪 improved faith - Differences in organisational culture 🡪 lack of trust 🡪 lack of task accomplishment - Willingness by actors 🡪 increased trust and faith 🡪 reduced conflict - Geographical coterminosity 🡪 increased inter-personal communication 🡪 enhanced cultural integration |  |
| (Idel, 2003) | Merger | Efficiency | - Staff involvement in transition 🡪 improved faith 🡪 improved task accomplishment | - Rejection of changes brought about by partnership 🡪 reduced faith 🡪 reduced task accomplishment - Perception of merger as a threat 🡪 reduced faith 🡪 reduced task accomplishment |  |
| (Jones, 2020) | Primary care network | Care quality |  | - Mutual understanding 🡪 improved trust 🡪 reduced conflict | Mandated |
| (Kershaw et al., 2018) | STP | Care quality | - Shared vision 🡪 increased trust 🡪 reduced conflict - Pre-existing relationships 🡪 greater initial level of trust 🡪 greater risk appetite - Clear vision 🡪 improved task accomplishment 🡪 increased trust - Lack of ‘natural geography’ that works for partners 🡪 enhanced task complexity 🡪 reduced faith | - Cross-sector partnering 🡪 greater cultural divide 🡪 greater task complexity - ‘champion’ leaders 🡪 improved trust 🡪 reduced conflict - Poor reputation of partnership type (STPs) 🡪 reduced faith 🡪 reduced uptake by stakeholders - Lack of agreement on process of partnership 🡪 unclear tasks 🡪 reduced task accomplishment - Regulation favouring competition and not supporting the partnership 🡪 increased task complexity 🡪 reduced faith | Mandated |
| (Lalani et al., 2018) | Quality improvement collaborative | Care quality/shared learning | - Task achievement 🡪 improved perception of progress 🡪 improved faith - Staff turnover 🡪 reduced faith 🡪 reduced collaborative behaviour - Lack of shared vision 🡪 reduced trust 🡪 reduced collaboration | - Clarity of purpose 🡪 improved faith 🡪 improved collaborative behaviour - ‘champion’ leaders 🡪 improved faith 🡪 more collaborative behaviour - Conflicting priorities 🡪 reduced perception of progress 🡪 reduced faith - Low faith 🡪 reduced cultural assimilation 🡪 increased conflict - Insufficient capacity 🡪 reduced faith 🡪 reduced collaborative behaviour - Positive reputation of the collaboration 🡪 improved initial faith 🡪 improved collaborative behaviour - Occurrence of inspections 🡪 increased initial faith 🡪 increased collaboration |  |
| (Leach et al., 2019) | Buddying | Care quality | - Regular meetings 🡪 improved communication 🡪 improved trust - Clear responsibilities 🡪 improved task accomplishment 🡪 improved trust | - Appoint champions 🡪 improved trust 🡪 reduced conflict | Mandated |
| (Lewis, 2005) | Primary care partnership | Care quality | - Legal agreements/ requirements to work together 🡪 greater interpersonal communication 🡪 greater trust | - Prior history of competition in locality 🡪 reduced trust 🡪 reduced desire to partner/increased conflict | Mandated |
| (Lim, 2014) | Merger | Efficiency | - Involvement of staff in process 🡪 improved faith 🡪 reduced conflict | - Effective management of staff expectations by leaders 🡪 improved faith 🡪 improved task accomplishment | Mandated |
| (Maniatopoulos et al., 2020) | Vanguards (eleven different cases) | Care quality | - Prior history of collaboration 🡪 increased initial trust 🡪 reduced chance of conflict - Overambitious aims 🡪 reduced task accomplishment 🡪 reduced trust | - Government focus on competition 🡪 increased task complexity 🡪 reduced faith - Insufficient funding 🡪 increased task complexity 🡪 reduced faith - Perceived competitiveness by partners 🡪 reduced trust 🡪 increased conflict - Cultural collision 🡪 reduced trust 🡪 increased conflict - Forming relationships prior to beginning the project 🡪 increased initial trust 🡪 reduced conflict - Conflict 🡪 additional work on ‘partnership functioning’ 🡪 reduced synergy/effectiveness - Workforce churn 🡪 greater need for interpersonal communication 🡪 continued trust-building - Cross-sector working 🡪 greater cultural divide 🡪 greater difficulty of cultural integration - Work focus diverted to partnership 🡪 reduced organisational performance 🡪 reduced faith in partnership - Unequal renumeration for involvement 🡪 reduced trust 🡪 increased conflict |  |
| (Mervyn et al., 2019) | Network | Care quality | - Geographical proximity 🡪 improved communication 🡪 improved trust | - Leaders perceived as uninterest 🡪 reduced faith 🡪 reduced synergy - Having supportive city culture 🡪 improved faith 🡪 improved synergy - Shared vision 🡪 improved progress 🡪 improved faith - Enthusiastic leader 🡪 improved faith 🡪 improved synergy - Transformational leadership 🡪 improved faith 🡪 improved synergy - Synergy 🡪 improved organisational learning from partners 🡪 improved effectiveness - Slow progress 🡪 reduced faith 🡪 reduced synergy |  |
| (Murray et al., 2018) | Accountable care organisation | Care quality |  | - Shared culture 🡪 improved trust 🡪 reduced conflict - Openly addressing tension 🡪 improved conflict reduction 🡪 improved trust - Constructive approach to conflict-resolution 🡪 reduced conflict 🡪 improved trust - Cultivation of perception of complementary expertise in partner 🡪 increased trust 🡪 reduced conflict - No visible return on investment 🡪 reduced faith 🡪 further reduced funding - Lopsided receipt of benefits of partnership 🡪 reduced trust 🡪 conflict |  |
| (Naylor et al., 2015) | Integrated care | Care quality | - Shared vision 🡪 increased trust 🡪 reduced conflict - Appropriate governance structure 🡪 clear accountability 🡪 improved trust - Clear outcomes, effective evaluation and review 🡪 improved task accomplishment 🡪 improved trust - History of joint working 🡪 improved trust 🡪 reduced conflict - Increased interpersonal communication 🡪 improved trust 🡪 reduced conflict | - Leadership based on shared accountability 🡪 reduced competitive behaviour 🡪 improved trust - Cross-sector working 🡪 more significant cultural divide 🡪 increased task complexity - Cross-sector leader appointments 🡪 reduced cultural divide 🡪 reduced task complexity - Resource pressure 🡪 return to competitive behaviours 🡪 reduced trust - Joint appointments 🡪 improved trust 🡪 reduced conflict - Contractually mandated risk-sharing 🡪 better conflict resolution 🡪 increased trust - Competition law 🡪 real or perceived barriers to partnership working 🡪 reduced faith |  |
| (NHS Employers, 2017) | Vanguards | Efficiency | - Prior history of competition 🡪 reduced trust 🡪 increased conflict - Prior reputation of organisation 🡪 trust 🡪 conflict - Proximity 🡪 greater frequency of communication 🡪 increased trust | - Collaborative behaviours 🡪 reduced trust 🡪 increased conflict - Perceived regulator instability 🡪 reduced faith 🡪 reduced uptake of partnership - Behavioural consistency 🡪 increased trust 🡪 reduced conflict - Cultural distance 🡪 trust 🡪 conflict - Fear of ceding control 🡪 competitive behaviour 🡪 reduced trust - Implementing ‘quick wins’ 🡪 task accomplishment 🡪 increased trust - Overpromising 🡪 not achieving tasks 🡪 reduced trust - Helping others out in difficult circumstances (leadership) 🡪 increased trust 🡪 reduced conflict - Inclusive decision-making 🡪 shared vision 🡪 increased trust - Showing evidence that collaboration will work 🡪 increased faith 🡪 reduced conflict - Formation of cross-partner teams 🡪 increased trust 🡪 reduced conflict - Documenting and sharing successes across partnership 🡪 increased trust 🡪 reduced conflict |  |
| (NHS Providers, 2019) | Integrated care | Care quality | - Prior history of collaborating 🡪 increased trust/faith 🡪 reduced conflict | - Obtaining agreement of employees to changes 🡪 increased faith 🡪 reduced conflict - Staff passport arrangements 🡪 increased communication 🡪 increased trust - Pooled resources 🡪 shared projects 🡪 increased trust - Legislative change to support system working 🡪 increased faith 🡪 reduced conflict - Cross-sector working 🡪 greater cultural divide 🡪 greater task complexity - Collaborating on staff recruitment 🡪 increased organisational cross-working/flexibility 🡪 increased trust - Place-based model of recruiting staff 🡪 more effective working patterns 🡪 improved task accomplishment/care quality |  |
| (NHS Providers, 2018) | Integrated care | Care quality | - Legal agreements in early phase (i.e. MoU) 🡪 greater initial trust 🡪 reduced conflict - Effective evaluation 🡪 improved task accomplishment 🡪 improved trust - Staff engagement and involvement 🡪 improved faith 🡪 reduced conflict | - Competitive behaviour 🡪 reduced trust 🡪 increased conflict - Extension of risk-management systems to system-wide risk 🡪 mutual accountability 🡪 increased trust |  |
| (NHS Providers & NHS Clinical Commissioners, 2018) |  |  | - Lack of shared purpose/vision 🡪 reduced trust 🡪 increased conflict - Engagement with patients and the public 🡪 focus on care quality 🡪 improved faith | - Collaborative leadership which transcends organisational boundaries 🡪 improved trust 🡪 reduced conflict - ‘Fortress mentality’ 🡪 reduced collaborative behaviour 🡪 reduced trust/faith - Legislation favouring competition 🡪 reduced collaborative behaviour 🡪 reduced trust/faith - (Leaders) not shying away from difficult conversations 🡪 improved trust 🡪 reduced conflict - (leaders) including all partners equally 🡪 improved trust 🡪 reduced conflict - Agreed-upon conflict resolution mechanisms/ accountability 🡪 improved conflict resolution 🡪 reduced conflict - Open book accounting 🡪 reduced competitive behaviour 🡪 increased trust - History of prior system failures 🡪 reduced initial trust & faith 🡪 reduced collaboration |  |
| (Peck et al., 2001) | Joint commissioning | Care quality | - Shared culture 🡪 improved trust 🡪 reduced conflict | - Co-location of staff 🡪 improved cultural integration 🡪 improved trust - Mutual understanding of culture 🡪 improved trust 🡪 reduced conflict - Cultural integration 🡪 improved trust 🡪 reduced conflict |  |
| (Pickup, 2004) | Integrated care/joint commissioning, mental health | Care quality |  | - Co-location of staff 🡪 greater interpersonal communication 🡪 greater cultural assimilation - Dedicated leadership team for managing partnership 🡪 improved task accomplishment 🡪 improved trust - Involvement of many partnership organisations 🡪 increased difficulty of communication 🡪 reduced coordination/task achievement - Equal participation of leadership of partners on shared board 🡪 improved trust 🡪 reduced conflict |  |
| (Round et al., 2018) | Integrated care | Care quality | - Overambition 🡪 reduced task achievement 🡪 reduced trust/faith - Lack of communication 🡪 reduced trust 🡪 increased conflict - Evaluation 🡪 increased task achievement 🡪 increased trust - Stakeholder involvement 🡪 improved faith 🡪 reduced conflict - Cuts to funding 🡪 increased task complexity 🡪 decreased task accomplishment - Unclear outcome measures 🡪 lack of task accomplishment 🡪 reduced trust/faith |  |  |
| (Shaw, 2002) | Mergers | Efficiency | - History of competition 🡪 reduced trust 🡪 increased conflict | - Strong culture 🡪 reduced intra-organisational conflict 🡪 improved trust - Preexisting strong cultures 🡪 mutual distrust 🡪 increased conflict - Perception of external interference 🡪 reduced faith 🡪 reduced task accomplishment - ‘Open’ rather than ‘closed’ organisational culture/leadership style 🡪 improved collaborative attitude 🡪 increased trust - Staff turnover 🡪 reduced faith 🡪 increased conflict - Unclear base of authority 🡪 reduced task accomplishment 🡪 reduced faith - Empathetic approach to managing staff reaction to merger process 🡪 improved faith 🡪 reduced conflict | Mandated |
| (Smith et al., 2020) | Primary care network | Care quality | - Clear roles for leaders 🡪 improved shared vision 🡪 improved perception of progress - Clear communication 🡪 improved progress 🡪 improved faith - Shared goals 🡪 improved progress 🡪 improved faith - Incompatible IT systems 🡪 increased task complexity 🡪 reduced faith - History of prior collaborations 🡪 improved initial trust 🡪 improved synergy/reduced conflict | - Leaders empathetic to staff 🡪 improved faith 🡪 reduced workforce turnover - Having a single ‘collaboration champion’ 🡪 improved trust 🡪 reduced conflict - Lack of strong leadership 🡪 lack of progress 🡪 lack of faith - Having dedicated operational managers for partnership 🡪 improved progress 🡪 improved faith - Bigger differences in ethos, culture, and procedures 🡪 increased task complexity 🡪 reduced progress - Fear of loss of organisational autonomy 🡪 reduced initial faith 🡪 reduced faith - Lack of staff engagement 🡪 reduced cultural assimilation 🡪 reduced trust - Only partial inclusion of partners involved in prior collaborations and new collaborations 🡪 distrust 🡪 reduced synergy - Lack of policy focus on rural networks 🡪 increased task complexity 🡪 reduced faith - Difficulty recruiting staff 🡪increased task complexity 🡪 reduced faith - High standards of collaborative specification 🡪 reduced ability for small practices to meet requirements 🡪 increased task complexity | Mandated |
| (Southby & Gamsu, 2018) | Integrated care, primary care networks | Care quality | - Regular communication 🡪 increased trust 🡪 reduced conflict - Common aims 🡪 increased trust 🡪 reduced conflict - Mutual respect 🡪 increased trust 🡪 reduced conflict - Physical proximity 🡪 increased trust 🡪 reduced conflict - Lack of resource 🡪 increased task complexity 🡪 reduced task accomplishment - Cultural distance 🡪 increased task complexity 🡪 reduced faith - Staff turnover 🡪 reduced faith 🡪 increased conflict - Stakeholder involvement 🡪 increased faith 🡪 reduced conflict | - Negative view of partnership type 🡪 reduced faith 🡪 reduced task accomplishment - Lack of regulatory support 🡪 increased task complexity 🡪 reduced faith - Poor reputation with commissioners 🡪 reduced faith 🡪 reduced synergy |  |
| (Southwark and Lambeth Integrated Care, 2016) | Integrated care | Care quality | - Shared vision 🡪 increased coordination 🡪 increased trust - Co-creation with citizens 🡪 increased legitimacy of arrangement 🡪 increased faith - Effective evaluation 🡪 greater task achievement 🡪 increased faith/trust - Overambition 🡪 reduced perception of achievement 🡪 reduced faith/trust - Strong governance & accountability 🡪 improved conflict resolution 🡪 reduced conflict | - Leadership churn 🡪 lack of vision 🡪 reduced trust/faith - Lack of agreed outcomes 🡪 reduction in ability to evaluate 🡪 reduced perception of task accomplishment - Use of evidence to underline vision 🡪 improved faith 🡪 reduced conflict - Use of robust business case with well-calibrated ambitions 🡪 increased task accomplishment 🡪 increased faith/trust - Competitive behaviour 🡪 reduced trust 🡪 increased conflict |  |
| (Starling, 2018) | Vanguards | Care quality | - Building upon pre-existing relationships 🡪 increased initial trust 🡪 reduced conflict - Starting small 🡪 building trust from initial successes 🡪 reduced conflict | - Use of logic models 🡪 improved shared understanding of partnership 🡪 improved faith |  |
| (Steininger et al., 2016) | Hospital merger | Care quality | - Sufficient capacity 🡪 increased perception of progress🡪 increased faith | - Absence of decision-making personnel 🡪 reduced faith 🡪 reduced collaborative behaviour |  |
| (Timmins, 2019) | Integrated care | Care quality |  | - Absence of legal structure for new organisation 🡪 reduced decision-making 🡪 reduced task achievement - Reduced financial resource 🡪 increased task complexity 🡪 reduced task achievement - Robust change management strategy 🡪 improved task achievement 🡪 reduced performance loss |  |
| (The King’s Fund, 2005) | Joint commissioning | Virtuousness | - Task achievement 🡪 increased faith 🡪 reduced conflict - Early winners 🡪 increased trust & faith 🡪 reduced conflict - Staff turnover 🡪 reduced faith 🡪 increased conflict - Involving stakeholders 🡪 increased faith 🡪 reduced conflict - Clear roles for involved organisations and teams 🡪 improved task accomplishment 🡪 improved faith/trust - Interpersonal communication 🡪 improved trust 🡪 decreased conflict - Geographical coterminosity 🡪 improved communication 🡪 improved trust | - Clear vision 🡪 focused work 🡪 increased task achievement - Leadership turnover 🡪 reduced clarity of vision 🡪 reduced task accomplishment - Having a leader dedicated to partnership work 🡪 offloading of ‘partnership work’ 🡪 increased partnership functioning - Shared financial resource 🡪 increased trust 🡪 reduced conflict - Limited financial resource 🡪 increased task complexity 🡪 reduced faith |  |
| (Timmins, 2019) | Integrated care | Care quality |  | - Empathetic leadership 🡪 improved faith & trust 🡪 reduced conflict - Evidence based change 🡪 increased faith 🡪 reduced conflict - Stable leadership 🡪 clear vision 🡪 increased faith - Legislation pushing for competition 🡪 increased task complexity 🡪 reduced faith - Optimistic leaders 🡪 improved faith 🡪 reduced conflict - Analytical skills 🡪 improved evaluation 🡪 improved task accomplishment - Poor reputation of partnership type (STPs) 🡪 reduced faith 🡪 reduced uptake by stakeholders - Lack of statutory powers 🡪 inability to appoint official systemwide leaders 🡪 reduced authority of partnership - Regulatory mandate to partner 🡪 reduced intrinsic motivation 🡪 reduced faith |  |
